# Supplementary material for: Splice-Junction-Based Mapping of Alternative Isoforms in the Human Proteome
Source: Cell Rep. Author manuscript; Available in PMC 2020 Jan 15. (PMC6961840; doi:10.1016/j.celrep.2019.11.026)
Supplement: 2 [file NIHMS1546469-supplement-2.zip › DF1/PXD010154/AdrenalGland/AdrenalGland_22_EIF4A1_DIETFYNTSIEEMPLNVADLMSASQDSRTEPNPEERPR.pdf]

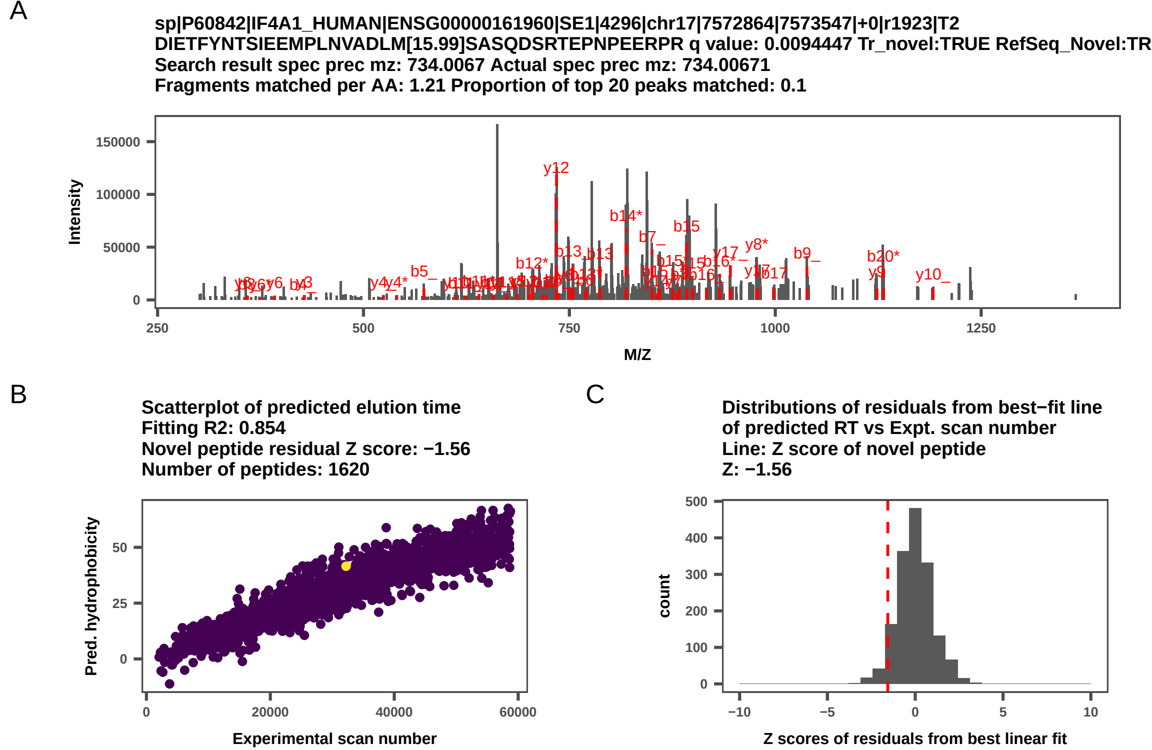
A
sp|P60842|IF4A1_HUMAN|ENSG00000161960|SE1|4296|chr17|7572864|7573547|+0|r1923|T2
DIETFYNTSIEEMPLNVADLM[15.99]SASQDSRTEPNPEERPR q value: 0.0094447 Tr_novel:TRUE RefSeq_Novel:TR
Search result spec prec mz: 734.0067 Actual spec prec mz: 734.00671
Fragments matched per AA: 1.21 Proportion of top 20 peaks matched: 0.1
Intensity
150000
100000
50000
0
250
500
750
1000
1250
M/Z
B
Scatterplot of predicted elution time
Fitting R2: 0.854
Novel peptide residual Z score: −1.56
Number of peptides: 1620
Pred. hydrophobicity
50
25
0
0
20000
40000
60000
Experimental scan number
C
Distributions of residuals from best−fit line
of predicted RT vs Expt. scan number
Line: Z score of novel peptide
Z: −1.56
count
500
400
300
200
100
0
−10
−5
0
5
10
Z scores of residuals from best linear fit
